# A Neurovisceral Integrative Study on Cognition, Heart Rate Variability, and Fitness in the Elderly

**DOI:** 10.3389/fnagi.2020.00051

**Published:** 2020-03-06

**Authors:** Felipe de Oliveira Matos, Amanda Vido, William Fernando Garcia, Wendell Arthur Lopes, Antonio Pereira

**Affiliations:** ^1^Departament of Physical Education, Health Sciences Center, State University of Maringá (UEM), Maringá, Brazil; ^2^Institute of Technology, Federal University of Pará (UFPA), Belém, Brazil

**Keywords:** cognition, executive functions, working memory, autonomic nervous system, heart rate variability, aging, physical fitness

## Abstract

The association between physical fitness and cognitive performance has been widely investigated in the literature. However, the neurophysiological mechanisms underlying this relationship are not yet clear. Here, we aim to evaluate the interactions between executive function measures, heart rate variability (HRV), and physical fitness in the context of the neurovisceral integration (NVI) theory. Twenty-eight healthy elderly subjects (>60 years) were submitted to evaluation of executive performance with three computerized tests: the N-back test measured working memory capacity, the Stroop Color test evaluated inhibitory control and selective attention, and the Wisconsin Card Sorting Test (WCST) evaluated abstract reasoning and cognitive flexibility. We also used the Physical Testing Battery for the Elderly to measure aerobic capacity, dynamic balance, upper body flexibility, and handgrip strength. Our results confirm the relationship between executive function and physical fitness, particularly between working memory, cardiorespiratory fitness, and dynamic balance. We also demonstrate an association between executive performance and HRV in older people, corroborating previous results from other groups obtained in young adults. However, our regression models did not indicate that HRV mediates the relationship between cognition and physical fitness in the elderly, suggesting that age-related degeneration of autonomic control can affect aspects of NVI in this population.

## Introduction

The neurovisceral integration (NVI) model (Thayer and Lane, 2000; Thayer et al., 2009) proposes that adaptive behavior depends on the integration of neural networks spanning both the central (CNS) and autonomic nervous systems (ANS) tasked with regulating cardiovascular function. Brain metabolism critically depends on the cardiovascular supply of cerebral blood flow (CBF) due to the limited availability of this organ’s intracellular energy substrates. The crosstalk between CNS and ANS structures, necessary to provide the brain with adequate levels of oxygen and energy sources, is mediated by a network of brain areas known as the “central autonomic network” (CAN; Benarroch, 1993; Valenza et al., 2019), which includes the anterior cingulate cortex (ACC), insula, and the ventromedial prefrontal cortex (vmPFC). Outputs from these regions eventually reach premotor neurons located in the lower brain stem and nucleus ambiguus in the medulla oblongata which contributes to the sympathetic and parasympathetic modulation of the heart (Thayer and Lane, 2000; Benarroch, 2012).

The heart rate variability (HRV) reflects the variation in the time interval between consecutive heartbeats (Saul, 1990; Task Force of the European Society of Cardiology and the North American Society of Pacing and Electrophysiology, 1996) and has been considered a surrogate parameter for the complex interaction between brain and cardiovascular system (Ernst, 2017). Both systems are simultaneously affected by the normal process of aging (Peters, 2006; Jandackova et al., 2016) and the result is a decline in the capacity of the CAN to adjust the CBF in response to environmental challenges while negatively influencing emotion control (Mather and Thayer, 2018) and cognitive performance (Thayer et al., 2009; Elias and Torres, 2017; Forte et al., 2019). Jandackova et al. (2016) showed a marked decline in HRV in middle-aged individuals which seems to be secondary to other pathological conditions. Cognitive dementia is the result of multiple age-related neuropathies (Power et al., 2018) associated with cardiovascular risk factors (Oishi et al., 2017), such as chronic brain hypoperfusion (Zheng et al., 2019), which particularly affects the frontal cortex (Sherwood et al., 2011).

Over the past decade, a large number of studies have investigated the role lifestyle activities play in decreasing the risk of cognitive decline in the elderly (for review, see Christie et al., 2017). Among these activities, physical exercise has gained prominence due to its neuroprotective effects against dementia and other neurodegenerative diseases (Phillips et al., 2014; Vecchio et al., 2018). For instance, research has shown that regular exercise promoted hippocampal neurogenesis (Erickson et al., 2009, 2011), local increases on the concentration of neurotrophins (such as BDNF; Leckie et al., 2014; Håkansson et al., 2017), neuroplasticity (Erickson and Kramer, 2009; Voss et al., 2013a,b; Erickson et al., 2014), angiogenesis (Bloor, 2005; Al-Jarrah et al., 2010), and adaptive changes in CBF (Dupuy et al., 2015; Jennings et al., 2015). Electrophysiological and neuroimaging studies have provided evidence that cardiorespiratory fitness is positively correlated with brain function, particularly in brain regions associated with the CAN, which has shown increased neuroplasticity after physical exercise interventions, improving both cardiovascular and cognitive control (Gomez-Pinilla and Hillman, 2013). However, relatively few studies have focused on the relationship between physical fitness and effects on NVI biomarkers such as HRV, particularly in the elderly (see Albinet et al., 2010, 2016; Dupuy et al., 2018). While Alderman and Olson (2014) demonstrated the role of physical fitness in improving autonomic and neurocognitive health in young adults, these authors failed to show HRV-mediated influences between cardiorespiratory fitness and cognitive performance, suggesting that other mediators may be more relevant in this population, which is at the apex of cognitive performance and with not much individual difference in cardiorespiratory fitness, compared to the elderly. In addition, other studies that investigated the relationship between cognition, HRV, and fitness from an NVI perspective have focused exclusively on aerobic capacity as an independent variable (Alderman and Olson, 2014; Dupuy et al., 2015).

Thus, the main purpose of the current study is to fill the gap in the literature regarding the association of physical fitness with cognitive performance and HRV in elderly subjects. Besides, we aim to verify how this relationship is associated with different physical abilities, such as strength, flexibility, dynamic balance, and aerobic capacity.

## Materials and Methods

### Participants

Twenty-eight (28) subjects (24 women) aged 60 years and over (mean age 66.71 ± 7.64 years old) participated in the research. The volunteers were screened using the following inclusion criteria: being over 60 years old, be literate, possess normal or corrected visual acuity, possess familiarity with computers, and have medical clearance to perform physical activities.

The exclusion criteria were: smoking (<6 months), surgery (<6 months), official medical diagnosis of psychiatric, psychological or cardiac disease, use of medication that may impair cardiac autonomic control and/or cognitive functions, Mini-Mental State Examination (MMSE) score <24 (or <21 for subjects with lower schooling levels; Almeida, 1998), Geriatric Depression Scale-Short Form (GDS-SF) score >5.

All experimental procedures performed in this study were approved by the Research Ethics Committee of the State University of Maringá (1,161,402).

### Outcome Measures

#### Cognitive Measurements

We used three tests to evaluate different dimensions of executive function, namely the N-back test (working memory), the Stroop task (selective attention and inhibitory control), and the Wisconsin Card Sorting Test (WCST; abstract reasoning and cognitive flexibility). All tests were performed in a computerized setup created with the software Presentation Version 20.2 (Neurobehavioral Systems, Inc., Berkeley, CA, USA).

#### The Wisconsin Card Sorting Test (WCST)

The WCST (Grant and Berg, 1948) is a problem-solving task for cognitive performance assessment. The test consists of matching test cards one by one with stimulus cards, following a rule that must be deduced by the subjects themselves. We used 64 test cards which had to be associated with four stimulus cards (1-one red triangle; 2- two green stars; 3- three yellow crosses; and 4- four blue circles). The match is made by pressing on the keyboard the number corresponding to the desired stimulus card. After each attempt, the participant received positive or negative feedback on their performance through the words “right” or “wrong” presented on the screen. Blocks of 10 or 11 stimuli were presented and at the end of each one, the rule changed. We used as a parameter to evaluate performance the total number of errors and the number of perseverative errors. These scores were chosen because they are sensitive to the effects of aging (MacPherson et al., 2002; Guarino et al., 2019).

#### Stroop Color

We used the Victoria version of the Stroop test (Spreen and Strauss, 1998), which consists of 72 stimuli, distributed in three tasks with 24 items each. The task is divided into three blocks, starting after an instruction presented on the screen explains the task to be performed in each step. The stimuli are presented for 2 s and if no response is given within 5 s another stimulus is presented.

The first block was composed of colored rectangles in the colors green, pink, blue and brown; the second block consisted of neutral words (each, never, today, everything) written with the colors of the previous rectangles; the third block had stimuli with a word-color conflict. At each stimulus presented the subjects should press a key with a color corresponding to the stimulus presented on the screen.

We used response latency as a measure of performance.

#### N-back

We used a spatial n-back task adapted from Vermeij et al. (2014). The test has three levels, 0-back (control condition), 1-back (low working memory load) and 2-back (high working memory load). The test consisted of black square-shaped stimuli, which could appear at any one of 14 fixed locations on a monitor screen. Before each stimulus, a fixation point was presented at the center of the screen. Each trial lasted 2,500 ms and consisted of 2,000 ms of fixation and 500 ms of stimulus presentation. The task began after the subjects pressed a key indicating they had read the instructions on the screen. Each level consisted of 60 trials, with 17 target stimuli that should be identified by the subject. During instruction a screen containing the black squares in all possible positions appeared for 30 s, then a quick presentation of each stimulus was made for 500 ms in a sequence from left to right and bottom to top on the screen.

Test performance was evaluated by the percentages of 2-back hits, total hits, and misses.

#### Heart Rate Variability

Heart Rate was recorded with the subjects at rest in a comfortable and quiet room for 10 min. R-wave peaks from the ECG were recorded continuously using a Polar HRM V800 heart rate monitor (Polar OY, Finland) with a sampling rate of 1,000 Hz for subsequent analysis of HRV. R-R intervals (the interval between two successive R waves of the ECG’s QRS signal) were visually inspected for ectopic beats and replaced by interpolated data from adjacent normal to normal (N-N) intervals. A period of 5 min with lowest variance was selected for analysis. We performed both time and frequency domain analysis of the ECG signal with the Kubios software (Kubios Oy). In the time domain, we calculated the mean HR, the standard deviation of the N-N intervals (SDNN) and the square root of the successive quadratic mean interval differences (RMSSD), which are the most common measures representing parasympathetic activity (Task Force of the European Society of Cardiology and the North American Society of Pacing and Electrophysiology, 1996). In the frequency domain, we used Fourier transform to quantify spectral density power of low frequency (LF; 0.04–0.15 Hz) and high frequency (HF; 0.15–0.40 Hz) bands. Additional calculations included LF + HF, LF and HF expressed in normalized units and the LF/HF ratio. We used the Poincaré graph to quantify SD1 and SD2 (Task Force of the European Society of Cardiology and the North American Society of Pacing and Electrophysiology, 1996).

#### Physical Assessment

To assess physical abilities we used a functional fitness battery test for elderly people (Rikli and Jones, 1999a). Aerobic capacity was measured by the distance covered in meters during a 6-min walk test (6MWT). For measures of agility, speed, and dynamic balance, we used the Timed Up-and-Go test (TUG), in which the subjects should sit in a chair, stand up, walk to a cone 2.44 meters away, return, and sit again in the shortest time possible (time was measured in seconds). Flexibility was evaluated with the sit and reach test, measured in centimeters. Additionally, Handgrip strength was measured using a hand dynamometer with the dominant hand (North Coast Medical, Inc., Morgan Hill, CA, USA). Three trials from the dominant hand were calculated and the median was used for the analysis (Innes, 1999). Handgrip strength is expressed in kilograms (kg).

#### Data Collection Procedures

The evaluations were performed in three moments along 2 days. In moment 1, the participants signed the Informed Consent Form and answered the MMSE and GDS-SF questionnaires. Then, they went through a familiarization period with the computerized cognitive tests. Moments 2 and 3 occurred 1 week later to minimize learning effects on the performance of cognitive tests. In moment 2 we recorded the subjects’ resting heart rate (HR) before they undertook tests of executive function. The moment 3 occurred soon after and consisted of physical fitness measures.

#### Statistical Analysis

We performed an exploratory analysis with the Shapiro–Wilks test. For variables with normal distribution, we used the Pearson correlation test and, for non-normal distributions, we used the Spearman correlation test. Finally, multiple linear regressions with the hierarchical method were used for the independent variables, in which the researchers chose the order of the variables inserted in the model. We used the Durbin–Watson test for the independance of residuals. We also tested for multicollinearity and no strong correlation coefficients were found between the independent variables. There were no outliers in the residual statistics (with standard deviations below −3 and +3). We used the SPSS 21.0 Software (IBM Corp.) for analysis.

## Results

Table 1 presents the results of the descriptive analysis of cognitive, HRV and physical variables. Correlations between cognitive measures and HRV parameters are shown in Table 2. We found significant correlations between the number of misses in the working memory task (N-back) and both mean HR (*r* = 0.406, *p* = 0.032) and mean RR intervals (*r* = − 0.383, *p* = 0.044). As for the WCST results, the number of misses correlated significantly with the following HRV indexes, RMSSD (*r* = 0.476, *p* = 0.012), LF (n.u.; *r* = −386, *p* = 0.046), HF (%; *r* = 0.426, *p* = 0.027), HF (n.u.; *r* = 0.389, *p* = 0.045), SD1 (*r* = 0.473, *p* = 0.013), and SD2/SD1 ratio (*r* = −0.465, *p* = 0.014). The performance on the Stroop test did not correlate with any of the HRV variables.

**Table 1:** Descriptive values of cognitive performance, heart rate variability (HRV), and fitness.

	Mean (±SD)	Median	Minimum	Maximum
**HRV**
Mean HR	78.18 (9.12)	77.50	59	98
Mean RR	777.21 (94.43)	772	613	1018
SDNN	17.94 (16.22)	12.55	7.8	81.5
RMSSD	21.16 (26.12)	13.55	4.6	116.8
LF (%)	49.62 (17.31)	53.15	10.10	77.45
LF (n.u.)	55.84 (20.13)	58.68	10.14	92.70
HF (%)	40.01 (20.02)	33.14	5.54	89.14
HF (n.u.)	43.82 (19.96)	39.86	7.26	89.49
Total Power	394.29 (906.52)	119.50	30	4781
LF/HF	2.06 (2.44)	1.48	0.113	12.77
SD1	14.99 (18.49)	9.55	3.3	82.7
SD2	19.76 (14.71)	15.700	8.0	80.4
SD1/SD2	1.77 (0.77)	1.70	0.68	3.98
**Physical variables**
6MWT (m)	504.11 (78.7)	497.5	350	675
TUG (ms)	7,371.4 (2244.9)	6,595	4,970	15,600
Strength (Kg/f)	25.71 (8.7)	24	14	47
Flexibility (cm)	3.8 (9.7)	5.7	−22	18
**Cognition**
**N-back**
Hits 2-back	49.02 (21.9)	47.10	0.0	82.4
Hits N-back	67.75 (13.5)	70.60	44.1	91.2
Miss N-back	16.65 (8.4)	15.80	2.5	37.5
**Stroop**
latency C_A (ms)	8,414.99 (22,329.81)	1,100.4	−8,838.3	84,365.6
latency C_B (ms)	1,538.90 (7414.47)	−69.0	−14,215.3	26,546.8
latency RT C_A (ms)	98.90 (223.83)	62.9	−248.9	910.8
latency RT C_B (ms)	101.86 (243.77)	47.6	−110.8	1,109.0
**Cards of Wisconsin**
Miss	31.26 (7.52)	31.00	15	52
Perseverative Miss	9.59 (3.70)	10.00	4	16

**Table 2:** Correlations between HRV and cognitive parameters.

	Mean HR	Mean RR	SDNN	RMSSD	LF (%)	LF (n.u.)	HF (%)	HF (n.u.)	Total power	LF/HF	SD1	SD2	SD2
**N-back**
Hit 2-back	−0.050	0.075	−0.023	0.082	−0.026	0.019	−0.022	−0.022	−0.070	−0.037	0.085	−0.087	−0.101
Hit N-back	−0.115	0.128	0.070	0.132	−0.090	−0.016	−0.009	0.016	0.004	−0.094	0.132	−0.019	−0.117
Miss N-back	0.406*	−0.383*	0.123	0.246	−0.204	−0.250	0.258	0.255	0.012	−0.256	0.248	0.010	−0.189
**Stroop**
Latency C_A	0.153	−0.118	0.264	0.130	0.024	−0.027	0.054	0.031	0.182	−0.022	0.124	0.217	0.009
Latency C_B	−0.166	0.164	0.082	0.296	−0.052	−0.125	0.165	0.131	−0.018	−0.123	0.292	−0.050	−0.242
Latency RT C_A	−0.207	0.218	0.217	0.193	−0.008	−0.051	0.065	0.050	0.129	−0.045	0.188	0.142	−0.070
Latency RT C_B	−0.244	0.244	0.137	0.285	−0.037	−0.071	0.106	0.080	−0.069	−0.071	0.283	0.008	−0.226
**Cards of Wisconsin**
Misses	0.109	−0.095	0.277	0.476*	−0.302	−0.386*	0.426*	0.389*	0.229	−0.379	0.473*	0.127	−0.465*
Perseverative Misses	0.106	−0.103	0.067	0.229	−0.095	−0.175	0.209	0.170	0.074	−0.192	0.228	0.011	−0.340

*p < 0.05.

**Figure 1:**
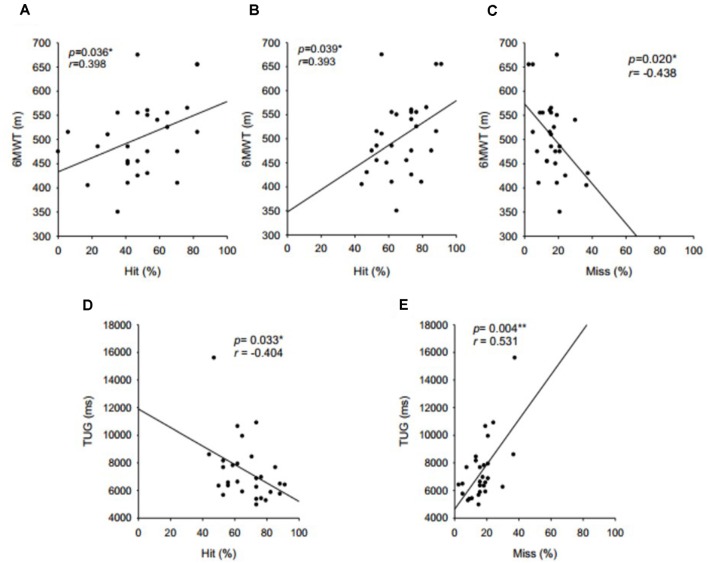
Correlations between working memory, aerobic capacity, and dynamic balance. **(A)** Hits in 2-back level and walk, **(B)** total hits in N-back test and walk, **(C)** misses in N-back and walk, **(D)** total hits in N-back test and TUG, and **(E)** misses in N-back and walk.

Working memory performance measured by the N-back correlated positively with aerobic performance (6MWT) and dynamic balance (TUG; Figure 1). Only the number of hits on the 2-back test did not show a significant correlation with the TUG (*r* = −332, *p* = 0.084). No significant correlation was found between working memory and either handgrip strength or flexibility. The other cognitive tests also showed no significant correlation (*p* < 0.05) with physical fitness variables.

Table 3 shows the correlations measures between HRV indexes and physical fitness parameters. No significant correlation (*p* < 0.05) was found between these variables in the present study.

**Table 3:** Correlation between HRV and physical parameters.

	6MWT	TUG	Strength	Flexibility
Mean HR	−0.198	0.018	−0.173	0.071
Mean RR	0.215	−0.014	0.172	−0.099
SDNN	0.058	0.068	−0.301	0.058
RMSSD	0.148	0.130	−0.227	0.148
LF (%)	−0.027	−0.044	−0.134	0.151
LF (n.u.)	−0.057	−0.012	−0.121	0.044
HF (%)	0.090	−0.007	0.097	−0.018
HF (n.u.)	0.058	0.025	0.119	−0.043
Total power	0.025	−0.014	−0.153	0.018
LF/HF	−0.074	−0.015	−0.118	0.069
SD1	0.157	0.123	−0.218	0.138
SD2	0.031	−0.003	−0.231	−0.054
SD1/SD2	−0.164	0.054	0.063	−0.150

*no correlation was statistically significant*.

### Multiple Regression Model for Mean HR

We analyzed the impact of cognition variables (independent variables) N-back misses and C-A Latency on the HRV index and the Mean HR (dependent variables). Regarding the correlation coefficients between the independent variables, an acceptable variation was verified regarding the absence of multicollinearity (correlation coefficient ranging from −0.327 to 0.406). The Durbin-Watson’s diagnosis of independent residue verification was 2.458 and confirmed the quality of the model.

We observed that both models had moderate correlation coefficients (0.406 < *r* < 0.530), and the coefficients values increased by including more independent variables to the model (Model 2). Similarly, *R*^2^ indicates that the percentage change in the dependent variable explained by the independent variables increased as they were input into the model (0.165 < *R*^2^ < 0.281), suggesting that model 2, associated with N-back misses and AC latency in the Stroop test account for approximately 28% of the variation in mean HR. The change statistics revealed that the construction of the two models sequentially improved the model prediction, indicating statistical significance for the insertion of an independent variable in relation to a model without predictors (*p* = 0.032), improving the model by approximately 16% (*p* = 0.016; Table 4).

**Table 4:** Impact analysis, change statistics and analysis of variance for models involving mean HR and cognition.

			Change statistics			ANOVA	
Models	*r*	*R*^2^	*R*^2^	*F*	*sig* F	*F*	*p*
**1**	0.406	0.165	0.165	5.144	0.032*	5.144	0.032*
**2**	0.530	0.281	0.116	4.039	0.049*	4.892	0.016*

*Model 1: mean HR (dependent variable) × Miss Nback (independent variable). *Model 2: mean HR (dependent variable) × Miss Nback Latency C-A (independent variable). *P* < 0.05*.

Finally, ANOVA confirms that the fit of the model with predictors is better than the fit without predictors (*p* < 0.032).

Table 5 shows that the regression coefficients of both models have a significant impact on the understanding of the relationships between the independent and dependent variables. Model 2 shows that the significance values are all below 0.049, confirming the relevance of the variables N-back misses and C-A Stroop Latency for the model. It is noteworthy that the standardized coefficients reveal that the variable “N-back miss” has greater relevance to the model (coefficient = 0.496) when compared to “C-A Stroop latency” (coefficient = 0.0352).

**Table 5:** Regression coefficient for models involving mean HR variability and cognition variables.

				Collinearity	
Models	Std.Coeff.	*t*	*sig*	Tolerance	VIF
1
Miss Nback	0.406	2.268	0.032*	1.000	1.000
2
Miss Nback	0.496	2.838	0.009*	0.936	1.096
Latency C-A	0.352	2.010	0.049*	0.936	1.069

*Model 1: mean HR (dependent variable) × Miss Nback (independent variable). *Model 2: mean HR (dependent variable) × Miss Nback Latency C-A (independent variable). *P* < 0.05*.

### Multiple Regression Model for Mean RR

We evaluated the impact of the cognitive variable “N-back misses” (independent variables) on the HRV variable “Mean RR” (dependent variable). Regarding the correlation coefficients between the independent variables, an acceptable variation was found regarding the absence of multicollinearity (correlation coefficient ranging from −0.383 to 0.045). The Durbin-Watson’s diagnosis was 2.419, confirming the quality of the model.

Table 6 presents the analysis of the correlation coefficients and impact of N-back misses on mean RR, as well as the change coefficients and the ANOVA value for the model. The correlation coefficient of the model was moderate (*r* = 0.383) and *R*^2^ (0.147) indicates that the percentage variation of mean RR explained by N-back was approximately 14%.

**Table 6:** Impact analysis, change statistics and analysis of variance for the model involving miss N-back and mean RR.

			Change statistics			ANOVA	
Model	*r*	*R*^2^	*R*^2^	*F*	*sig* F	*F*	*p*
1	0.383	0.147	0.147	4.472	0.044*	4.472	0.044*

*Model 1: mean_RR (dependent variable) × Miss Nback (independent variable). *P* < 0.05*.

Table 7 shows that the regression coefficient has a significant impact on the understanding of the relationships between the independent variable and the dependent variable.

**Table 7:** Regression coefficient for the model involving miss N-back and mean RR.

				Cofllinearity	
Model	Std.Coeff.	*t*	*sig*	tolerance	VIF
1
Miss N-back	−0.383	−2.115	0.044*	1.000	1.000

*Model 1: mean HR (dependent variable) × Miss N-back (independent variable). *P* < 0.05*.

## Discussion

The purpose of the present study was to evaluate how physical fitness contributes to executive performance in the context of the NVI model in elderly subjects. Normal physiological aging is known to increase the probability of the occurrence of pathological changes in the brain (Fjell et al., 2014) which are associated with different trajectories of cognitive performance in the elderly, including dementia (Sorond et al., 2015). Even though recent studies have shown the benefits of physical exercise and the positive influence of increased aerobic capacity on mitigating the deleterious effects of aging on cognitive measures (Kramer et al., 2006; Miller et al., 2012), many questions remain regarding the relative impact of different exercise modalities and the indirect role played by cardiovascular improvements on those measures. While a recent study Alderman and Olson (2014) demonstrated the positive role of aerobic capacity in both autonomic and cognitive measures in young adults, the authors were not able to show a correlation between these outcomes with changes in HRV, suggesting that brain-cardiovascular interactions may not be the most important mediators of change in this population.

The physical fitness of our sample was within the expected values for their age, when compared to normative values available in the literature (for references see: Innes, 1999; Rikli and Jones, 1999b; Mazo et al., 2015). Our subjects also performed comparably to other reports in the literature that used the same working memory tasks (Vermeij et al., 2014). The only exception was the number of hits in the N-back test, which was considerably lower, probably due to cultural specificities and the low educational level of our sample when compared to other studies. Our results also corroborated previous findings on the relationship between cognitive performance and physical fitness (Hansen et al., 2004; Erickson and Kramer, 2009; Erickson et al., 2009; Albinet et al., 2010; Chang et al., 2012; Soares-Miranda et al., 2014; Håkansson et al., 2017; Northey et al., 2018). Specifically, we demonstrated that working memory is associated with the performance in the 6-min walk and TUG tests, which are reliable indicators of aerobic capacity and dynamic balance in the elderly (Figure 1). These results are in agreement with other studies that showed a direct correlation between aerobic capacity and performance on tasks relying on executive functions (Kramer and Erickson, 2007; Alderman and Olson, 2014; Dupuy et al., 2015, 2018). Hansen et al. (2004) found that after a period of 8 weeks of aerobic training individuals not only improved performance on cognitive tasks (working memory, sustained and selective attention), but that this result was accompanied by improvements in HRV. The authors then performed a detraining period and found that this manipulation caused a reduction in both cognitive performance and HRV, indicating that they were associated with physical fitness. However, another study that investigated the cognitive performance of athletes throughout the sport season found that at times of large increased physical demand there was an associated increase in simple reaction time (slower cognitive processing) accompanied by reduced athletic performance, which indicated a dose-response relationship between exercise intensity and cognition (Matos et al., 2014). Surprisingly, these authors showed that changes in physical training did not induce significant changes in HRV, suggesting that other adjacent mechanisms may be involved in this interaction between cognition and physical capacity.

In our study, we also found a correlation between performance on cognitive tests and HRV (Table 2). For instance, there are positive correlations between N-back errors and mean HR and negative correlations between N-back errors and average RR intervals. This result indicates that higher parasympathetic activity is associated with better scores in working memory tasks. We also found a positive correlation between the number of errors in the WCST and heart indices associated with parasympathetic activity (RMSSD, percentage of HF, HF n.u and SD1) and a negative correlation between the number of errors with sympathetic activity (LF n.u. and SD2). This result leads us to consider that higher parasympathetic and lower sympathetic activity are associated with a decrease in behavioral flexibility. Other studies support the hypothesis that better performance in executive functions is associated with a higher parasympathetic activity as measured by HRV (Hansen et al., 2003; Duschek et al., 2009; Thayer et al., 2009). Apparently, this relationship between cognition and HRV is important for self-regulation under various behavioral conditions (Blasi et al., 2006; Thayer et al., 2009; Capuana et al., 2014; Mather and Thayer, 2018).

Regarding the hypothesis that HRV could act as a link between cognition and physical performance, none of our regression models showed a significant relationship between executive function and HRV-mediated physical fitness. These results reinforce the negative findings of a previous study that investigated whether the influence of cardiorespiratory fitness on cognition of young adults would be mediated by HRV (Alderman and Olson, 2014). The researchers attributed their findings to the low variability in the aerobic capacity of their young volunteers, indicating that studies in other age-groups could obtain different results. However, our results corroborated their findings, suggesting that other mechanisms not associated with the NVI model may be responsible for the relationship between cognition and physical fitness in the elderly. The list of possible mediators includes neurotrophin levels, such as BDNF, and changes in CBF due to regular physical exercise (Dinoff et al., 2016).

Cardiovascular adaptations that positively influence HRV are generally attributed in the literature to the practice of aerobic exercises (Carter et al., 2003). However, other training methods such as resistance training, are also shown to promote cognitive improvements in the elderly (Liu-Ambrose et al., 2010; Bherer et al., 2013; Kattenstroth et al., 2013; Gajewski and Falkenstein, 2016; Müller et al., 2017; Northey et al., 2018; Lin et al., 2019). Chang et al. (2012) reviewed the effect of resistance training on elderly cognition and proposed IGF-1-mediated increases in neurogenesis, vascular density and glucose utilization in the brain as possible mediators. In addition, BDNF and VEGF (vascular endothelial growth factor) are also suggested as possible targets for exercise-related brain adaptations, which would explain the absence of correlation between HRV and physical fitness shown in our study. Our results also showed a lack of relationship between strength capacity and cognitive performance, pointing to the need for further studies exploring this association.

Age-related impairments in cognition, especially executive functions, are also associated with reduced HRV, probably due to the detrimental effects of aging on vagal activity (Britton et al., 2008; Frewen et al., 2013; Forte et al., 2019). The vagal tank theory (VTT) proposed by Laborde et al. (2018), uses the metaphor of a “vagal tank,” the level of which is determined by changes in cardiac vagal control activity and determines the efficiency of self-regulatory adaptations to cognitive, social, and emotional challenges. According to the VTT, risk of abnormal HRV, which is a measure of cardiac vagal control, is the impaired ability to cope with physical and emotional demands in daily life activities. One alternative to counteract these effects would be to engage in regular physical exercise, which improves both the basal “tank” volume and vagal reactivity (Hottenrott et al., 2019).

Even though it is widely known that physical activity improves cognitive and brain functions, there is still no consensus on the magnitude of its effects and which are the best strategies and adequate dosage (duration, intensity, and frequency; Kramer et al., 2005; Hillman et al., 2008; Erickson et al., 2019). Physical activity can promote either nonspecific or specific cognitive improvements, depending on the type of exercise, with executive functions being the most benefited (Gajewski and Falkenstein, 2016; Erickson et al., 2019). According to the Physical Activity, Cognition, and Brain Outcomes of the American College of Sports Medicine, training programs involving different components such as endurance, strength and coordination, from moderate to vigorous intensity over the long term, are the most adequate to provide cognitive benefits to older populations (>50 years; Erickson et al., 2019).

In summary, our results are consistent with the literature regarding the relationship between cognition and physical fitness. Our results also support the NVI model, now tested in elderly individuals, demonstrating the association between executive function performance and HR. However, the hypothesis of HRV acting as a link between cognition and physical fitness was not confirmed in our sample, which indicates that age-related degeneration of autonomic control can affect aspects of NVI in this population.

## Data Availability Statement

The datasets generated for this study are available on reasonable request to the corresponding author.

## Ethics Statement

The studies involving human participants were reviewed and approved by Permanent Committee on Human Research Ethics—COPEP, State University of Maringá. The patients/participants provided their written informed consent to participate in this study.

## Author Contributions

FM and AP designed the study and wrote the manuscript. AV and FM collected the data. AV, FM, WG, WL, and AP analyzed the data. All authors read and commented on the manuscript.

## Conflict of Interest

The authors declare that the research was conducted in the absence of any commercial or financial relationships that could be construed as a potential conflict of interest.
